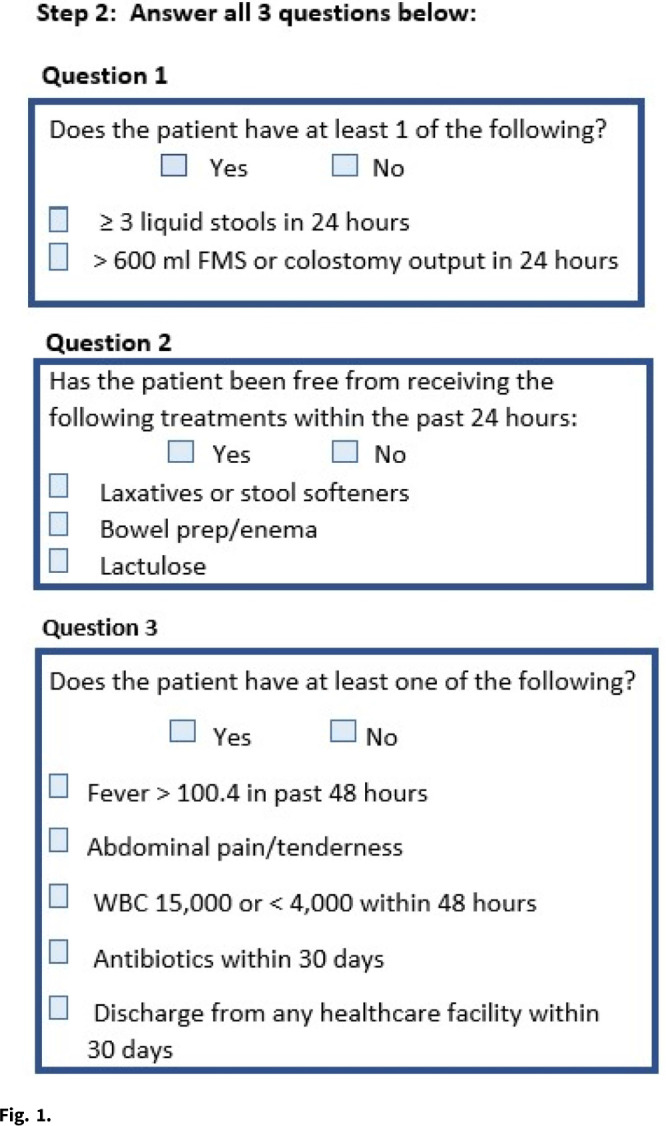# Prospective audit and feedback of *Clostridioides difficile* PCR at the time of ordering increases appropriateness of testing

**DOI:** 10.1017/ash.2022.109

**Published:** 2022-05-16

**Authors:** Daniel Tassone, Matthew Hitchcock, John Markley, Michael Stevens

## Abstract

**Background:** Over-testing for *Clostridioides difficile* infection outside acute diarrheal illness without a clear alternative cause can lead to inappropriate diagnosis and treatment with antibiotic therapy. Preanalytical interventions such as education, order restriction, and electronic order assistance are common but are limited in effectiveness. As an alternative approach, our antibiotic stewardship program (ASP) implemented prospective audit and feedback (PAF) on *C. difficile* PCR orders to reduce inappropriate testing.

**Methods:** The study was conducted at a 399-bed, tertiary-care, Veterans’ Affairs Medical Center and included adult inpatients and outpatients for whom *C. difficile* PCR testing was ordered. In the preintervention period from June through September 2019, the ASP was alerted to *C. difficile* PCR tests and collected data but did not intervene. From October 2019 to January 2020, the ASP performed real-time PAF at the time of ordering. Appropriateness of testing was determined based on whether there was a negative result in the prior 7 days and a 3-step review of clinical factors (Fig. 1). When possible, a direct conversation took place with the ordering provider. If not possible, a general note delineating appropriate clinical criteria for testing was generated. No PAF was done outside standard hours. The ASP recommended cancelling tests deemed inappropriate. Monthly test rates during the pre- and postintervention periods were compared using the Student *t* test with α = .05, and test appropriateness was compared using the χ^2^ test. All analyses were conducted using Microsoft Excel software. **Results:** During the preintervention period, a total of 418 tests were ordered (104.5 per month). This number decreased to 276 (69 per month) during the intervention period. (p **Conclusions:** Direct PAF at the time of *C. difficile* PCR ordering may increase test appropriateness and is associated with a reduction in overall testing, primarily by reducing the number of tests that are considered not appropriate on clinical grounds. PAF is effective but requires significant time investment by ASP staff and may not be a sustainable intervention over time.

**Funding:** None

**Disclosures:** None

**Figure:**